# KRAS mutation in relation to HER2 overexpression/amplification in colorectal cancer

**DOI:** 10.1186/1897-4287-10-S4-A25

**Published:** 2012-12-10

**Authors:** Julia K Bar, Anna Lis-Nawara, Arleta Lebioda, Anna Jonkisz, Michał Jeleń, Tadeusz Dobosz

**Affiliations:** 1 Department of Pathomorphology and Oncological Cytology, Wroclaw Medical University, Wroclaw, Poland; 2 Molecular Techniques Unit, Department of Forensic Medicine, Wroclaw Medical University, Wroclaw, Poland

## 

The development of targeted therapies for KRAS, EGFR and HER2 may increase the range of response in patients with colorectal cancer. Mutation at codons 12 and 13 of the KRAS gene has been shown to be predictive of cetuximab response in colorectal cancer. However, due to the combined effects of multiple oncogenes involved in disease progression of patients with colorectal cancer, it seems to be important to identify the molecular factors that characterize therapy-resistance phenotype of tumors.

Forty six paraffin-embedded colorectal cancer specimens were analyzed for KRAS mutation and HER2 overexpression/amplification. A high–resolution melting (HRM) assay and single-nucleotide polymorphisms (SNPs) were used to detect somatic mutation in exon 2 notably condons 12 and 13 of the KRAS gene. HER2 overexpression was detected using monoclonal antibody and confirmed by fluorescence in situ hybridization (FISH) analysis.

KRAS mutations for codons 12 and 13 were identified in 16/46 (34.7%) of patients by SNP. The alterations in KRAS gene were observed in similar percentage of both codons. Colorectal cancer showed mainly heterozygous 35G>A and 38G>A KRAS gene mutations.

HRM analysis showed presence of KRAS exon 2 mutation in 13/46 (28.2%) colorectal cancers. Despite positive SPN results in three cases , HRM technique did not reveal KRAS gene alteration. The concordance rate between the two methodologies was high at 87.5%. KRAS mutation was more frequently observed in poorly differentiated tumors and adenocarcinomas than in other histological types.

HER2 overexpression was found in 37/46 (80.3%) of all colorectal cancers and in 62.1%, 61.5% of KRAS mutation-positive cases detected by SNP and HRM techniques respectively. HER2 overexpression was accompanied by amplification of HER2 gene. The subgroup of colorectal cancers with KRAS mutation and HER2 overexpression/amplification was poorly differentiated.

In summary, the presence of HER2 overexpression/amplification in KRAS mutation–positive colorectal cancers suggests a possible role for the use of specific tyrosine kinase inhibitors in the treatment of disease.

